# Effective Use of the Thoracolumbar Interfascial Plane Block With Total IV Anesthesia for Laminoplasty in a Patient With Myasthenia Gravis: A Case Report

**DOI:** 10.7759/cureus.102532

**Published:** 2026-01-29

**Authors:** Shota Tanimoto, Tomoharu Shakuo, Yutaro Yamazaki, Yumi Umetani, Atsunori Sakamoto, Kenji Shida

**Affiliations:** 1 Anesthesiology, Showa Medical University Northern Yokohama Hospital, Yokohama, JPN

**Keywords:** lumbar laminoplasty, myasthenia gravis, opioid-sparing analgesia, thoracolumbar interfascial plane block, total intravenous anesthesia

## Abstract

Myasthenia gravis (MG) is an autoimmune disorder of the neuromuscular junction that causes fluctuating muscle weakness and fatigue. The development of myasthenic crisis and respiratory compromise presents significant challenges in the perioperative management of these patients. Surgical pain-induced stress is a substantial risk factor for myasthenic crisis. A 68-year-old man with MG underwent L3-L5 lumbar laminoplasty under total IV anesthesia. A bilateral ultrasound-guided thoracolumbar interfascial plane block (TLIPB) was performed at the L4 level following anesthesia induction. Intraoperative opioid administration was intentionally minimized, and postoperative analgesia was provided using IV patient-controlled analgesia (IV-PCA) with fentanyl. Within 24 hours, 10 boluses (total fentanyl dose: 250 μg) were administered, and the Numerical Rating Scale (NRS) score decreased to 3 by 20 hours postoperatively, at which time IV-PCA was discontinued. The patient exhibited no respiratory deterioration or signs of myasthenic crisis and was discharged on postoperative day 9. Achieving adequate analgesia while minimizing the risk of respiratory depression is critical in the perioperative management of patients with MG. In this case, TLIPB was successfully implemented as part of a multimodal analgesic strategy combined with total IV anesthesia to facilitate an opioid-sparing approach, with postoperative pain managed using IV-PCA and scheduled non-opioid analgesics, without any respiratory compromise.

## Introduction

Myasthenia gravis (MG) is an autoimmune disorder of the neuromuscular junction, characterized by fluctuating skeletal muscle weakness caused by autoantibodies targeting the postsynaptic membrane [1]. The onset of myasthenic crisis and respiratory depression poses significant challenges in the perioperative management of patients with MG. Surgical pain-induced stress is a notable risk factor for myasthenic crisis [2]. Although opioid analgesia provides effective pain control, it carries potential adverse effects, including respiratory depression. Furthermore, lumbar spinal surgery is often associated with substantial postoperative pain, highlighting the need for appropriate pain management strategies.

Herein, we report successful pain management in a patient with MG undergoing L3-L5 lumbar laminoplasty using an adjunctive thoracolumbar interfascial plane block (TLIPB), incorporated as part of a multimodal analgesic strategy to support an opioid-sparing approach. The TLIP block involves injecting a local anesthetic into the interfascial plane between the multifidus and longissimus muscles to block the dorsal rami of the thoracolumbar nerves. This technique attenuates pain arising from the posterior spinal elements and paraspinal muscles and is typically used for postoperative analgesia following posterior-approach lumbar spine procedures such as lumbar laminectomy or laminoplasty.

## Case presentation

A 68-year-old man (167 cm tall, weighing 65 kg) was referred to our hospital with a chief complaint of low back pain radiating to both buttocks. The patient reported acute-onset pain approximately one month prior, triggered by prolonged walking and unrelieved by oral medical treatment. His medical history included nasopharyngeal carcinoma treated with chemotherapy and radiotherapy, followed by sudden right-sided sensorineural hearing loss; resection of a tongue tumor under general anesthesia without perioperative complications; a diagnosis of MG; and, most recently, left retinal vein occlusion resulting in near-complete vision loss in the left eye. MG symptoms were stabilized with pyridostigmine (60 mg/day). Detailed MG treatment and evaluation records were managed at another hospital and were unavailable. Current oral medications included tramadol hydrochloride/acetaminophen combination tablets (two tablets/day), prochlorperazine maleate (10 mg/day), pregabalin (250 mg/day), clonazepam (0.5 mg/day), loxoprofen (180 mg/day), rebamipide (300 mg/day), rosuvastatin (2.5 mg/day), and pyridostigmine.

**Figure 1:**
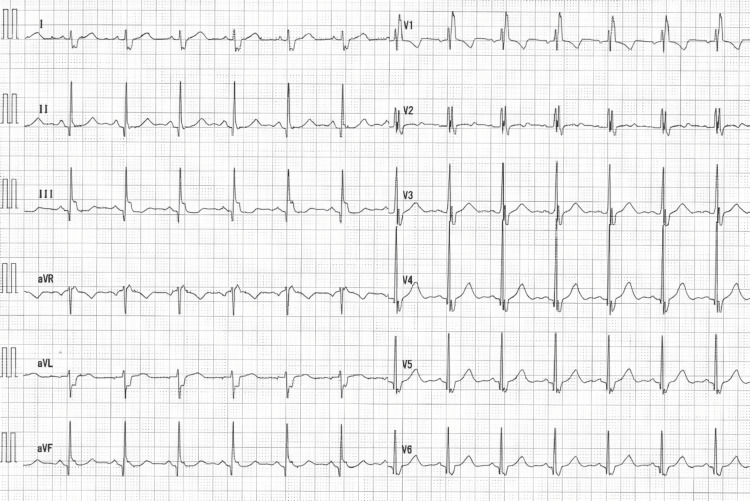
Electrocardiography

Preoperative physical examination revealed pain with a Numerical Rating Scale (NRS) score of 2 during movement, difficulty with trunk extension, and numbness in both buttocks. No MG-related symptoms, such as bulbar paralysis, were observed. The patient had no history of myasthenic crisis and no thymoma. Imaging studies revealed severe lumbar spinal canal stenosis, leading to a diagnosis of lumbar spinal stenosis. Blood tests showed elevated blood urea nitrogen (21 mg/dL), aspartate aminotransferase (46 U/L), and creatine kinase (582 U/L); all other parameters were within normal limits (Table 1). Electrocardiography revealed a complete right bundle branch block (Figure 1). Pulmonary function tests demonstrated a vital capacity of 3.00 L (78.1%), forced expiratory volume in 1 s (FEV1) of 2.18 L, and FEV1/forced vital capacity ratio of 80.9%, indicating mild restrictive ventilatory impairment. Laminoplasty of the L3-L5 vertebrae was planned.

**Table 1:** Blood test results ALB, albumin; ALP, alkaline phosphatase; ALT, alanine aminotransferase; APTT, activated partial thromboplastin time; AST, aspartate aminotransferase; BUN, blood urea nitrogen; Ca, calcium; CK, creatine kinase; Cl, chloride; CRE, creatinine; D-Bil, direct bilirubin; eGFR, estimated glomerular filtration rate; γ-GT, gamma-glutamyl transferase; Hb, hemoglobin; Ht, hematocrit; INR, international normalized ratio; K, potassium; LDH, lactate dehydrogenase; MCH, mean corpuscular hemoglobin; MCHC, mean corpuscular hemoglobin concentration; MCV, mean corpuscular volume; Na, sodium; PLT, platelet count; PT, prothrombin time; T-Bil, total bilirubin; T-Cho, total cholesterol; UA, uric acid

Parameter	Result	Unit	Reference range
WBC	6,970	/µL	3,500-9,700/µL
RBC	4.74	×10⁶/µL	4.38-5.73 × 10⁶/µL
Hb	15.5	g/dL	13.6-18.3 g/dL
Ht	45.9	%	40.4-51.9%
MCV	97	fL	83-101 fL
MCH	32.8	pg	28.2-34.7 pg
MCHC	33.8	%	31.8-36.4%
PLT	20.6	×10⁴/µL	14.0-37.9 × 10⁴/µL
PT (activity)	95.7	%	≥80%
PT (seconds)	11.3	seconds	10.0-13.0 seconds
INR	1.02	-	0.84-1.14
APTT	31.8	seconds	26.0-38.0 seconds
Total protein	7.8	g/dL	6.5-8.2 g/dL
ALB	4.6	g/dL	3.8-5.2 g/dL
BUN	21	mg/dL	8.0-20.0 mg/dL
CRE	0.84	mg/dL	0.65-1.09 mg/dL
eGFR (estimated)	70.2	-	-
UA	5.7	mg/dL	3.6-7.0 mg/dL
Na	137	mEq/L	135-145 mEq/L
K	4.3	mEq/L	3.5-5.0 mEq/L
Cl	100	mEq/L	98-108 mEq/L
Ca	9.2	mg/dL	8.6-10.2 mg/dL
T-Bil	0.18	mg/dL	≤1.2 mg/dL
D-Bil	0.2	mg/dL	≤0.4 mg/dL
AST (GOT)	46	U/L	10-40 U/L
ALT (GPT)	27	U/L	5-45 U/L
γ-GT	21	U/L	≤79 U/L
LDH	243	U/L	120-245 U/L
ALP	55	U/L	38-113 U/L
CK	582	U/L	50-230 U/L
CRP	0.18	mg/dL	≤0.30 mg/dL
T-Cho	198	mg/dL	150-219 mg/dL

General anesthesia combined with a peripheral nerve block was administered. Anesthesia was induced using propofol target-controlled infusion (5 μg/mL), remifentanil (0.5 μg/kg/min), and rocuronium (50 mg), and maintained with a combination of air, oxygen, and propofol (2.4-3.0 μg/mL). Propofol was administered using target-controlled infusion with a Terufusion TCI pump (TE-371; Terumo, Tokyo, Japan) based on the Marsh pharmacokinetic model. The depth of anesthesia was continuously titrated to maintain a bispectral index between 40 and 60.

A potential-sensing neuromuscular monitor (AF-200 Series neuromuscular module, Nihon Kohden, Tokyo, Japan) was applied to the ulnar nerve before anesthesia induction. Fifty milligrams of rocuronium were administered during induction, and tracheal intubation was performed after confirming a train-of-four (TOF) count of zero. Reducing the intubating dose of rocuronium was considered; however, intubation under a reduced dose could worsen intubating conditions, potentially necessitating repeated laryngoscopy and airway manipulation, which may increase the risk of vocal cord injury and postoperative hoarseness. Additionally, this case was expected to require time for prone positioning and performing the nerve block in the prone position, during which airway-related adverse events needed to be avoided. Therefore, given that the patient had clinically asymptomatic and stable MG, a single standard intubating dose of rocuronium was administered.

Following tracheal intubation, the patient was placed in the prone position. Bilateral ultrasound-guided TLIPBs were performed at the L4 level using a high-frequency linear transducer (SonoSite SII™; Fujifilm, Tokyo, Japan) and an in-plane needle approach. To identify the target level, the probe was first placed in the paramedian sagittal plane around the intercristal (Jacoby) line to visualize the lumbar spinous processes, then translated laterally to the paraspinal region. It was moved caudally to identify the sacrum and subsequently advanced cephalad to confirm the L4 level. Following confirmation, the probe was rotated 90° to obtain a transverse view in which the spinous and transverse processes were visualized, and the paraspinal muscles (multifidus and longissimus) were identified. From a lateral-to-medial direction, the needle was advanced in-plane toward the interfascial plane between the multifidus and longissimus muscles.

**Figure 2:**
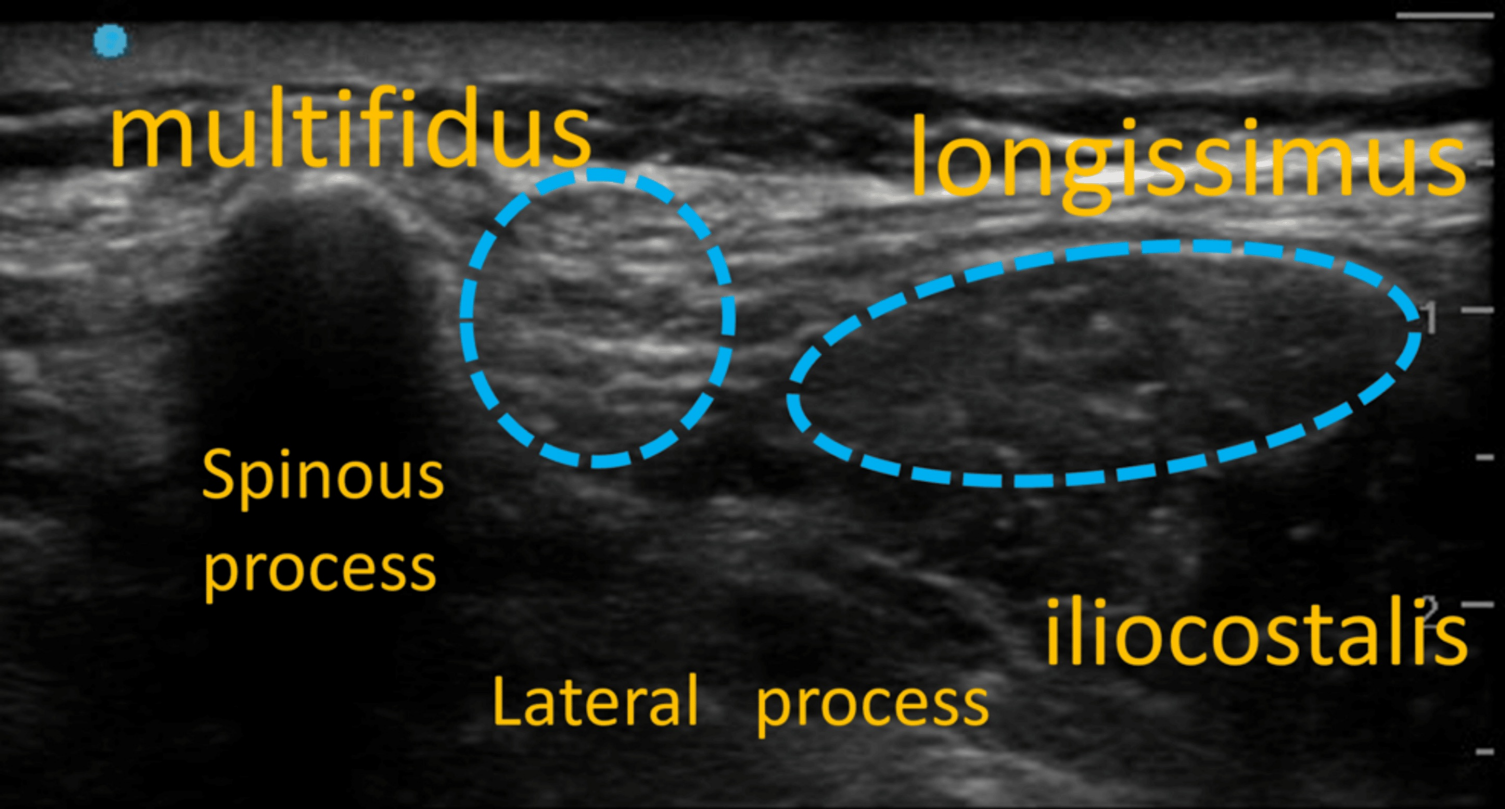
L4 ultrasound image before administering the TLIPB TLIPB, thoracolumbar interfascial plane block

**Figure 3:**
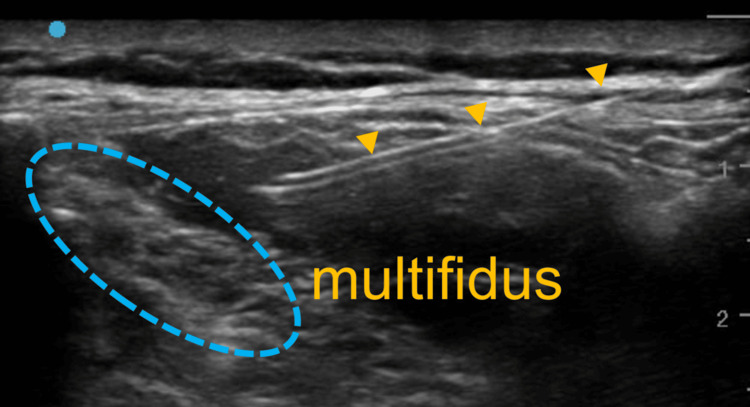
Imaging during TLIPB administration and local anesthetic injection The yellow triangles indicate the nerve block needle. TLIPB, thoracolumbar interfascial plane block

The TLIPB was performed after induction to avoid procedure-related distress and movement, allowing safe performance in the prone position with a secured airway. Given the potential perioperative risks of myasthenic crisis and opioid-induced respiratory depression, opioid administration was minimized, and pain management was planned using bilateral TLIPBs. After confirming the plane with saline hydrodissection, 0.25% levobupivacaine was injected (20 mL per side; total 40 mL). No additives were used (Figure 2, Figure 3).

IV patient-controlled analgesia (IV-PCA) was initiated intraoperatively using a continuous infusion pump (CADD-Solis^®^ PIB, Taiyo Nippon Sanso, Tokyo, Japan). The pump was programmed with a demand dose of 25 μg fentanyl, a lockout interval of five minutes, and no basal infusion (0 μg/h). This bolus-only setting was chosen because multimodal analgesia with a regional block can appropriately reduce opioid requirements; it minimizes fentanyl exposure while still allowing patient-triggered rescue dosing when needed.

Sugammadex (200 mg) was administered postoperatively. Extubation was performed after confirming a TOF ratio of ≥90% on two separate occasions and after establishing adequate spontaneous breathing. Quantitative TOF monitoring was performed from the preoperative period to objectively assess the depth of neuromuscular blockade and recovery. After administration of sugammadex, a TOF ratio of ≥90% was confirmed on repeated measurements to avoid residual neuromuscular blockade and prevent postoperative respiratory depression and airway complications, thereby mitigating perioperative risk in this patient with MG. Neuromuscular recovery after sugammadex was not prolonged compared with typical cases.

**Figure 4:**
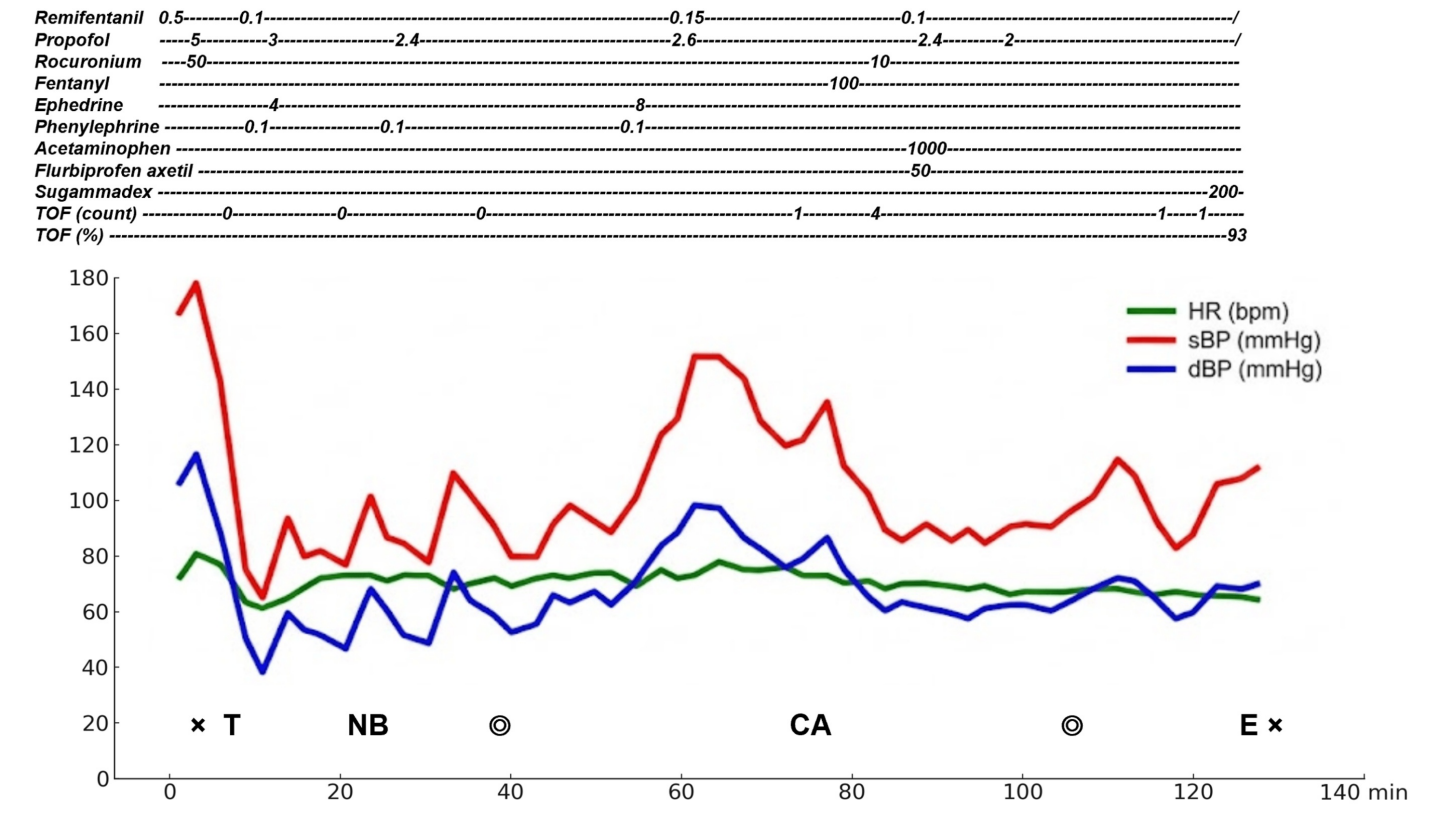
Anesthetic record Remifentanil (µg/kg/min); propofol (µg/mL); rocuronium (mg); fentanyl (µg); ephedrine (mg); phenylephrine (mg); acetaminophen (mg); flurbiprofen axetil (mg); sugammadex (mg) CA, start of IV patient-controlled analgesia; dBP, diastolic blood pressure; E, extubation; HR, heart rate; NB, nerve block; sBP, systolic blood pressure; T, tracheal intubation; TOF, train-of-four; x, start/end of anesthesia; ◎, start/end of surgery

The operative time was one hour and 14 minutes, and the total anesthesia time was two hours and 11 minutes (Figure 4).

Postoperatively, 1,000 mg of IV acetaminophen and 50 mg of flurbiprofen axetil were administered twice daily on a scheduled basis. A total of 10 IV-PCA bolus doses (total fentanyl: 250 μg) were delivered within 24 hours postoperatively. Of these, two were administered within the first 1.5 hours, six between two and eight hours, and one at 16 hours postoperatively. The NRS score was 1 immediately postoperatively, increased to 9 at 1.5 hours, remained at 6 from four to 10 hours, and decreased to 3 by 20 hours postoperatively, at which time IV-PCA was discontinued. At 16 hours postoperatively, the patient experienced no pain on ambulation.

Adequate pain control was achieved using a combination of IV-PCA and scheduled IV acetaminophen and flurbiprofen axetil, as reflected by the NRS decrease to approximately 3 by 20 hours postoperatively. NRS scores were assessed at rest after the patient returned to the ward, concurrently with routine vital signs monitoring by the nursing staff.

No respiratory deterioration or signs of myasthenic crisis were observed postoperatively, and pain scores improved over time as described. The patient demonstrated favorable recovery and was discharged on postoperative day 9. At our institution, the typical length of stay following lumbar decompression or laminoplasty is approximately 14 days, with most patients discharged within a range of five to 16 days. Therefore, discharge on postoperative day 9 was slightly earlier than usual but remained within the commonly observed range. No postoperative complications occurred, and pain control did not prolong hospitalization.

## Discussion

The prolonged effects of neuromuscular blockers and volatile anesthetics during anesthesia management [3] may precipitate perioperative myasthenic crisis and opioid-induced respiratory depression. The incidence of perioperative myasthenic crisis in patients with non-thymomatous MG is approximately 11% [4].

Myasthenic crisis, a life-threatening complication of MG, is defined as respiratory failure requiring invasive or noninvasive ventilation. Although it is often associated with weakness of the respiratory muscles, such as the diaphragm, it can also involve the palatal muscles, leading to upper airway collapse. Known triggers for myasthenic crisis include infection, surgical stress, adverse drug effects, complications, pregnancy, and reductions in immunosuppressive medication dosage [2]. However, the pathophysiology of myasthenic crisis remains incompletely understood due to the rarity of MG. Long-term predictors associated with myasthenic crisis and MG progression include disease severity at diagnosis, thymoma, and anti-MuSK antibody positivity [5]. Patients with MG exhibit increased opioid sensitivity [6], and surgical pain, particularly intense or prolonged pain, can potentially trigger myasthenic crises. Therefore, careful titration of prescribed analgesics is warranted to ensure adequate pain control while minimizing the risk of respiratory depression [7].

In this case, the patient had no history of myasthenic crisis, showed no bulbar symptoms, had no thymoma, and preserved vital capacity. Additionally, the disease was stable with a low dose of pyridostigmine, suggesting a low risk of postoperative myasthenic crisis.

Substantial pain was observed in the early postoperative period (peak NRS 9 at 1.5 hours), followed by decreasing analgesic demand over time. Ten IV-PCA boluses (total fentanyl: 250 μg) were administered within 24 hours. The final bolus was given at 16 hours, and IV-PCA was discontinued at 20 hours when the NRS decreased to 3, with no pain on ambulation. Notably, TLIPB was incorporated into a multimodal analgesic strategy alongside total IV anesthesia to support an opioid-minimizing approach. At our institution, lumbar decompression and laminoplasty procedures typically involve 100-200 μg of intraoperative fentanyl, with a total IV-PCA fentanyl dose of approximately 500 μg. In this case, IV-PCA was deliberately configured as bolus-only, with no basal infusion, to prevent inadvertent fentanyl over-administration caused by regional block effects and to limit opioid exposure to patient-triggered rescue dosing. Using this approach, the total fentanyl administered via IV-PCA within 24 hours was 250 μg, approximately half of the usual preparation for similar cases.

On the day of surgery, IV acetaminophen and flurbiprofen axetil were administered twice daily on a scheduled basis, although no standardized postoperative analgesic protocol is applied from the day after surgery onward. Despite the favorable outcome, this is a single case report without a comparator, limiting causal inference regarding the analgesic contribution of TLIPB and any opioid-sparing effect. Furthermore, as objective stress markers were not assessed, the effect of TLIPB and TIVA on perioperative stress cannot be determined. During a myasthenic crisis, postoperative progression of respiratory muscle or bulbar paralysis can occur within the first few days, potentially leading to respiratory arrest. Stress-induced postoperative crises following naloxone administration have been reported in suspected opioid overdose cases [8]. Peripheral nerve blocks are recommended for patients with MG, as they may reduce the need for intraoperative opioids and mitigate adverse effects on respiratory and gastrointestinal function. Oral cholinesterase inhibitors may be reinstated once adequate gastrointestinal function is established [9].

Nerve blocks applicable to lumbar spine surgery include TLIPB, modified TLIPB, and erector spinae plane block (ESPB). A recent study by Hand et al. reported the effectiveness of TLIPB [10]. In TLIPB, a local anesthetic is injected at the L3 level between the multifidus and longissimus muscles to block the dorsal branches of the thoracolumbar nerves. This technique provides analgesia from the midline to the thoracolumbar region by blocking posterior spinal nerve branches at a superficial level [10] and is typically used in surgeries involving two to three vertebral levels [11]. Its duration reportedly ranges from 2 to 24 hours, with evidence supporting its superiority over local infiltration at the surgical site [12]. In lumbar spine surgery, general anesthesia combined with TLIPB has been shown to reduce intraoperative and postoperative opioid requirements compared with saline controls, with significantly lower resting and activity pain scores at 24 hours postoperatively [13]. Similarly, in lumbar fusion surgery, general anesthesia plus TLIPB resulted in lower intraoperative remifentanil consumption and fewer IV-PCA doses than general anesthesia alone, with reduced visual analog scale scores and improved postoperative satisfaction [14].

Lumbar spinal surgery is associated with severe, diffuse postoperative pain [15] due to extensive manipulation of subcutaneous tissue, bones, and ligaments, which typically persists for at least three days [16]. Postoperative pain after lumbar laminoplasty is largely attributed to paraspinal muscle dissection and posterior element manipulation. TLIPB targets the dorsal rami by depositing local anesthetic between the multifidus and longissimus muscles, thereby attenuating pain from posterior structures and paraspinal muscles. Although multimodal analgesia, including preoperative and postoperative oral opioids, gabapentin, nonsteroidal anti-inflammatory drugs, and intraoperative ketamine, is frequently employed, local anesthetic techniques such as plexus blocks, facet joint blocks, and local infiltration at the surgical site are less commonly used. Compared with conventional nerve or plexus blocks, fascial plane blocks such as TLIPB may provide prolonged postoperative analgesia and reduce opioid requirements while minimizing motor weakness [14].

The modified TLIPB is an improved variant of TLIPB, involving injection of local anesthetic between the longissimus and iliopsoas muscles [15]. This approach avoids the midline, allowing clearer identification of anatomical structures and facilitating the technique [15]. In lumbar disc surgery, TLIPB and modified TLIPB provide comparable perioperative analgesic effects, including minimal responses to noxious stimuli, reduced pain scores, and decreased need for rescue analgesia, contributing to lower incidences of postoperative nausea and vomiting [16]. Both techniques demonstrate excellent opioid-sparing effects and safety in patients undergoing lumbar disc surgery [16]. Furthermore, several studies [17,18] comparing postoperative acute pain between ESPB and TLIPB in lumbar disc herniation surgery indicated that patients in the ESPB group required lower postoperative analgesic and opioid doses. However, compared with TLIPB, ESPB requires deeper injection of local anesthetic, which may delay therapeutic interventions for complications such as hematoma [14]. In this case, modified TLIPB may have contributed to effective back analgesia through a simpler nerve block technique extending beyond the surgical field.

## Conclusions

Attaining adequate analgesia while minimizing the risk of respiratory depression is crucial in the perioperative management of patients with MG. In this case, TLIPB was incorporated as part of a multimodal analgesic strategy to support an opioid-minimizing approach, with detailed reporting of postoperative analgesic requirements and respiratory outcomes. This approach enabled safe perioperative management, with no postoperative complications. The combined use of ultrasound-guided peripheral nerve blocks may be considered a feasible and safe strategy for intraoperative and postoperative care in patients with MG.
